# Improvement in Hemorrhoidal Disease Surgery Outcomes Using a New Anatomical/Clinical–Therapeutic Classification (A/CTC)

**DOI:** 10.1055/s-0040-1712542

**Published:** 2020-09-10

**Authors:** Gabriele Naldini, Filippo Caminati, Alessandro Sturiale, Bernardina Fabiani, Danilo Cafaro, Claudia Menconi, Domenico Mascagni, Felipe Celedon Porzio

**Affiliations:** 1 Proctology and Pelvic Floor Clinical Centre, University Hospital, Pisa, Italy; 2 Department of Surgical Sciences, Sapienza University of Rome, Rome, Italy; 3 Cirugia Coloproctologica, Hospital de la Fuerza Aerea de Chile, Santiago de, Chile

**Keywords:** hemorrhoids, hemorrhoid classification, hemorrhoidectomy, stapled hemorrhoidopexy, THD

## Abstract

**Introduction** The introduction and diffusion of new techniques for hemorrhoidal surgery have made it clear how much Goligher classification is inadequate in the modern times, lacking in any correlation between anatomical and clinical features to a surgical procedure. The aim of the study was to evaluate if the application of a new classification of hemorrhoidal diseases might lead to an improvement in the postoperative surgical outcomes.

**Methods** From January 2014 to December 2015, all patients undergoing surgery for hemorrhoidal disease were enrolled. The procedures performed were based upon a new anatomical/clinical–therapeutic classification (A/CTC) considering these items: anatomical presentation, symptom types and frequency, associated diseases, and available surgical treatments and their related contraindications. The new classification identified four groups: A (outpatient), B, C, and D (surgical approaches). The overall outcomes were assessed and then stratified by surgical groups. These data were then analyzed in comparison with the published data about all the surgical procedures performed.

**Results** A total of 381 patients underwent surgery and they were stratified as follows: Group B (39), C (202), and D (140). Group B underwent Doppler-guided dearterialization with mucopexies or tissue selective therapy, Group C stapled procedures, and Group D hemorrhoidectomy. The mean follow-up was 30 months. The overall outcomes were: success rate 92.4%, recurrences 7.6%, postoperative complications 4.8%, long-term complications 5.4%, and reoperation rate 2.7%. The success rates stratified by groups were: B, 85%); C, 91.4%; and D, 95.7%.

**Conclusion** The A/CTC proved to be useful in stratifying the patients and choosing the proper treatment for each case. This classification seems to improve the outcome of different surgical procedures if compared with those already published.

Hemorrhoidal disease (HD) is a very common anorectal disorder that affects millions of people around the world representing a major medical and socioeconomic problem. The literature about surgery for HD has tried to demonstrate the superiority of a specific technique compared with the others but treating a heterogeneous group of patients classified according to the Goligher classification. 1 2 Even though Goligher classification is the mostly spread and used, it has some relevant limitations such as: lack of prolapse quantification, lack of evaluation of symptom type, and frequency and the presence of any associated diseases. For these reasons, this classification does not appear useful for the treatment choice. Over the years, several classifications were proposed considering exclusively the symptoms, anatomical features, 3 4 or a specific score, 5 6 but none of them correlates to a specific treatment. Considering the overall outcomes of HD surgery, all the described techniques have their own pros and cons in terms of recurrence and reoperation rate, or early and long-term complications rate. 7 8 9 Conventional excisional surgery is both more clinically effective and less expensive if compared with circular stapled hemorrhoidopexy (CSH), which is associated with a shorter hospital stay, less pain, and earlier return to normal activities. 10 11 Indeed, considering all the surgical and outpatient options to treat HD, their advantages and disadvantages, the postoperative course, complications, and recurrence rates, the Authors built up a new anatomical/clinical–therapeutic classification (A/CTC) of HD trying to correlate the anamnestic data and clinical features with a specific surgical treatment.

The aim of the study was to evaluate if the application of this new A/CTC about HD in the clinical practice might lead to an improvement in the postoperative surgical outcomes.

## Methods

### Patient Selection

From January 2014 to December 2015, all patients with symptomatic HD undergoing surgical treatment were enrolled. All the data were recorded in a prospectively maintained database and then retrospectively analyzed. Those who did not have a scheduled visit with at least 24 months of follow-up were phone-called to get a proctologic reevaluation. The patients were preoperatively evaluated according to the following protocol: complete clinical evaluation with medical history, physical examination, and anoscopy. Those patients with impaired anal continence, with previous traumatic delivery, or prior proctological surgery were carefully evaluated through a preoperative anorectal manometry and 360° three-dimensional transanal ultrasound. On the contrary, those patients who referred obstructed defecation syndrome (ODS) underwent further preoperative investigations such as Rx-Defecography or dynamic pelvic magnetic resonance and anorectal manometry.

All the procedures performed were based upon the A/CTC, which was built up in 2013 based upon accurate literature review and decennial experience of high-volume specialized Proctology and Pelvic Floor Clinical Centre.

### Classification

The new A/CTC first considers the available surgical treatments and then, in order of importance for the procedure choice, it accounts the anatomical presentations, any possible contraindications related to the procedure, any associated diseases, and the types and frequency of symptoms. The possible treatments were classified in four groups as reported in Table 1 :

**Table 1:** New anatomical/clinical–therapeutic classification (A/CTC) of hemorrhoids

	Group A a	Group B	Group C	Group D
Treatment	RBL, IRC, sclerotherapy	Hemorrhoidal dearterialization and mucopexy	Tailored prolapse surgery with stapler (PPH, double PPH, high volume)	Excisional hemorrhoidectomy
Anatomy	Absent prolapse, very small prolapse	Small and asymmetrical prolapse, well-detectable hemorrhoidal peduncle	Circumferential prolapse (intraoperative evaluation)	Hemorrhoidal prolapse with large external piles
Relative contraindications	Stable external prolapse, intussusception	Stable external prolapse	Anal stenosis, impaired anal continence (absolute)	–
Associated disease	–	Anal fistula, fissure, impaired anal continence	ODS	Impaired anal continence, IBD, anal fistula, fissure, anal stenosis, coagulation disorders, anticoagulants and/or antiplatelets, immunotherapy
Type of symptoms	Bleeding, discharge	Bleeding (major symptom), discharge, continence disorders	Prolapse, bleeding, discharge	Acute hemorrhoidal edema, acute hemorrhoidal thrombosis, discharge
Frequency of symptoms	Frequently, always	Sometimes, frequently, always

Abbreviations: IBD, inflammatory bowel disease; IRC, infrared coagulation; ODS, obstructed defecation syndrome; PPH, procedure for prolapsed hemorrhoids; RBL, rubber band ligation.

a Nonsurgical patients not included in the study.

- Group A (outpatient treatments such as rubber band ligation [RBL], infrared coagulation, and sclerotherapy)
- Group B (hemorrhoidal artery ligation and mucopexy or tissue selective therapy [TST])
- Group C (CSH with low- and high-volume devices)
- Group D (hemorrhoidectomy)

Because Group A included outpatient approaches, it was excluded from the analysis that focused only upon surgical procedures (Groups B, C, and D).

**Fig. 1:**
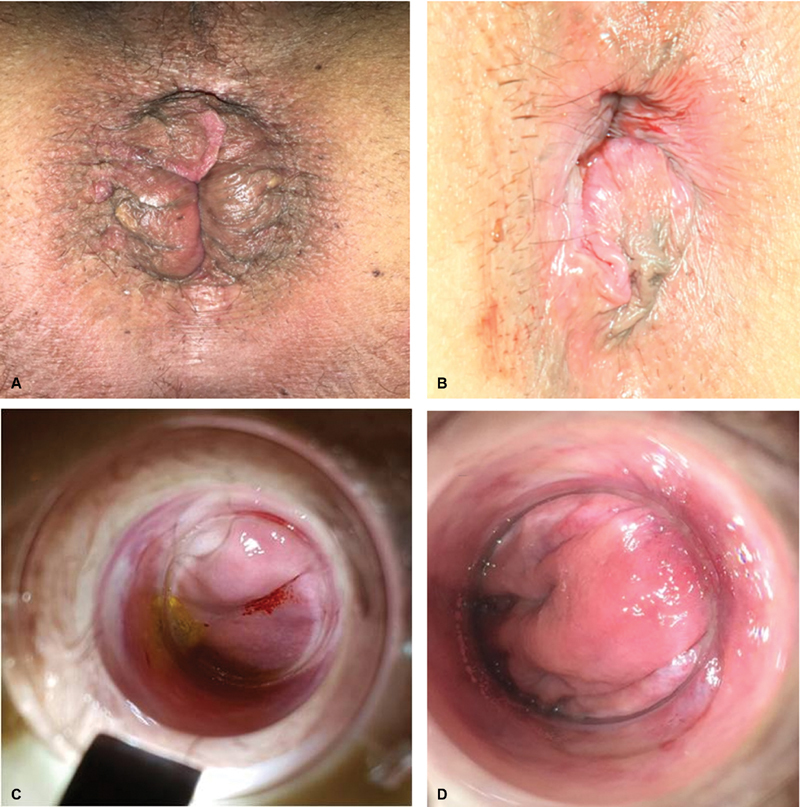
Group B: anatomical presentations. ( **A, B** ) Small prolapse; ( **C** ) internal asymmetrical prolapse; ( **D** ) well-detectable hemorrhoidal peduncle.

**Fig. 2:**
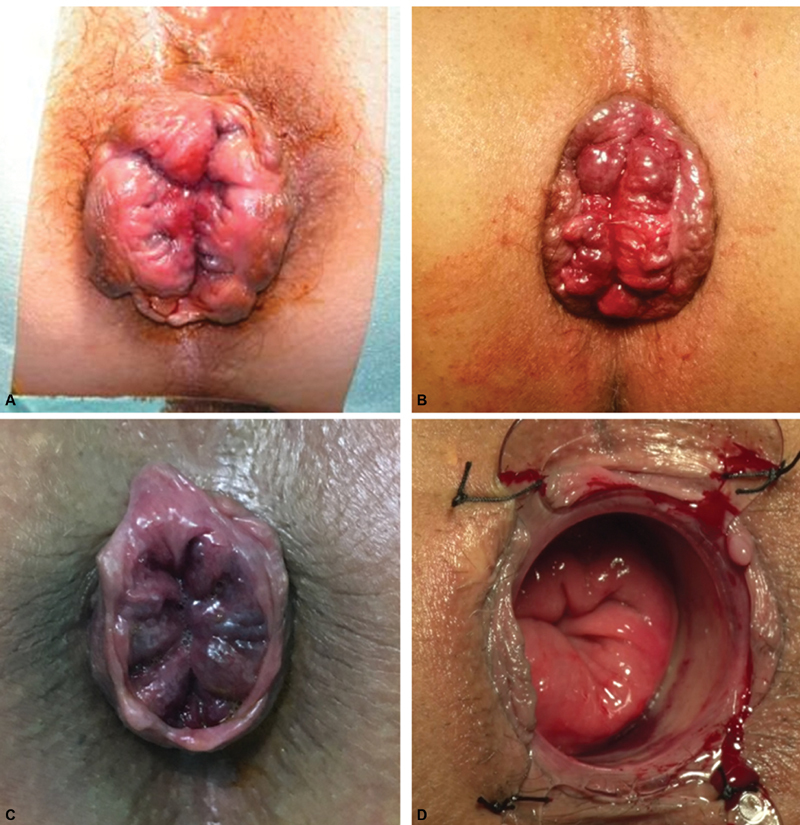
Group C: anatomical presentations. ( **A, B** ) Circumferential prolapse; ( **C, D** ) circumferential prolapse (intraoperative evaluation).

**Fig. 3:**
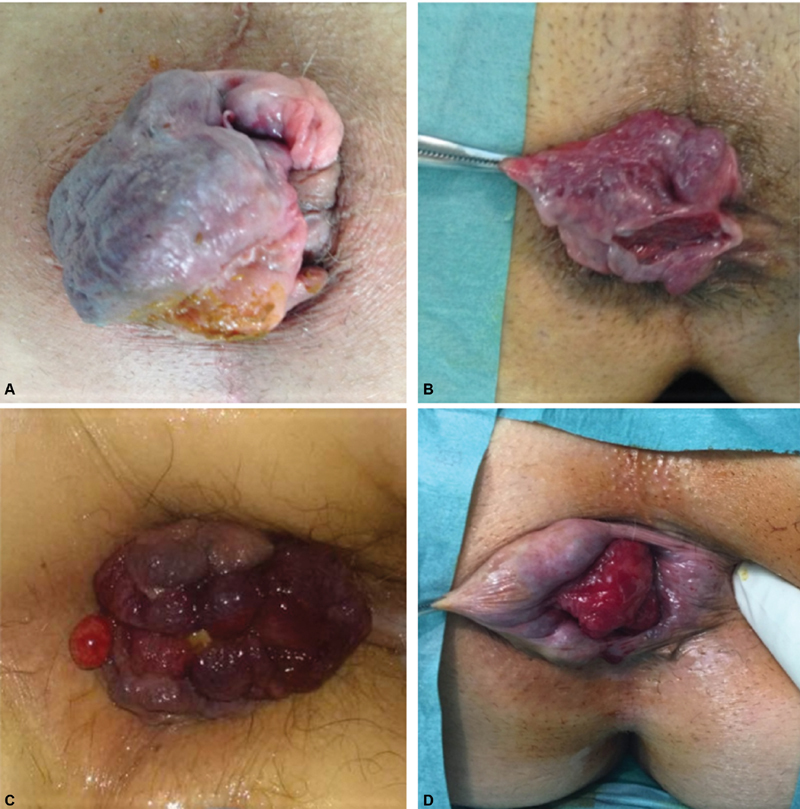
Group D: anatomical presentations. ( **A–D** ) Hemorrhoidal prolapse with large external piles.

The items analyzed were four:

1. Anatomical presentation: very small prolapse, small and asymmetrical prolapse, well-detectable hemorrhoidal pedicle, circumferential prolapse, and hemorrhoidal prolapse (HP) associated with large external piles ( Figs. 1 – 3 ).
2. Relative contraindications: stable external prolapse, recto-anal intussusception, anal stenosis, and impaired anal continence; some contraindications for a specific treatment may represent an indication for another one.
3. Associated disease: ODS, inflammatory bowel disease (IBD), anal fistula, fissure, coagulation disorders, immunotherapy, and anticoagulants and/or antiplatelets therapy.
4. Type and frequency of symptoms:
  ○ The types were: the presence of prolapse, bleeding, pain, thrombosis, acute hemorrhoidal swelling, or external prolapse and discharge.
  ○ The frequency of symptoms was divided as follows: rarely (less than three episodes per year), sometimes (more than three episodes per year), frequently (once a week), always present (everyday).

### Statistical Analysis

Comparison between groups was performed using the two-proportion *z* -test. A *p* -value < 0.05 was considered statistically significant.

## Results

A total of 381 patients (male [M] = 191; female [F] = 190]) were enrolled with a mean follow-up of 30 (range: 25–37) months.

### Patient and Group Characteristics

### Group B

The number of patients belonging to this group was 39 (10.2% [M = 14; F = 25]). Three patients (7%) underwent previous RBL. The main symptoms reported were bleeding (82%), pain (38%), burning (5%), and previous hemorrhoidal thrombosis (13%). The associated disorders were: impaired anal continence (28%), anal fissures (15%), anemia (4%), and symptomatic skin tags (4%). The operations performed were: (a) Doppler-guided dearterialization with associated mucopexies including transanal hemorrhoidal dearterialization (THD; 59%) and hemorrhoidal artery ligation with recto-anal repair (HAL-RAR) trilogy (31%), and (b) TST (10%). In 17.7% of the cases, there was an associated procedure such as sphincterotomy (7.6%), single pile hemorrhoidectomy (5.1%), and skin tags excision (2.5%).

### Group C

The number of patients was 202 (53% [M = 101; F = 101]). The previous proctologic procedures were hemorrhoidectomy (2.5%), CSH (2.5%), RBL (8%), and nonhemorrhoidal surgery (2%). The main symptoms were: prolapse (90%), bleeding (60%), discharge (25%), and pain (10%). The procedures performed were CSH with high-volume device (36 mm in diameter; 52.5%), CSH with procedure for prolapsed hemorrhoids (PPH; 33 mm in diameter; 40%), and double stapled hemorrhoidopexy (DSH; 7.5%). The associated procedures were: partial excision of the external hemorrhoid (14.8%) and skin tags excision (9.9%).

### Group D

Precisely, 140 patients (36.7%) belonged to this group (M = 76; F = 64). Previous proctologic surgeries were: hemorrhoidectomy (7%), HAL-RAR (1.4%), CSH (8%), cryotherapy (1.4%), DSH (0.7%), RBL (8%), and internal Delorme (0.7%). The main symptoms were: bleeding (76%), pain (42%), burning (9%), and previous hemorrhoidal thrombosis or edema (83%). In 43.5% of the patients, HD was associated to other proctologic diseases such as anal fissures (20%), impaired anal continence (12.5%), proctitis (6.4%), anemia (1%), and others (3.6%). The operation performed was an open hemorrhoidectomy with excision of three piles in 68.2% and two piles in 31.8%. The associated procedures were: mucopexy (10.8%), fissure treatment (6.4%), and fistula treatment (3.5%).

### Surgical Outcomes

The final results were analyzed considering recurrence and reoperation rates, and early as well as long-term complications for each group, as shown in Table 2 .

**Table 2:** Surgical results of the prospective study, with mean follow-up of 30 months

Rates	Group B (%)	Group C (%)	Group D (%)	Total (%)
Success	85	91.1	95.7	92.1
Early complications	2.5	3.4	6.4	4.4
Long-term complications	0	6.9	2.8	4.7
Reoperations	2.5	3.9	1.4	2.8

### Group B

The recurrence rate was 15% with a reoperation rate of 2.5%. Only one case (2.5%) of postoperative bleeding was treated conservatively. No long-term complications were recorded.

### Group C

The recurrence rate was 8.9% and it was subdivided as complete, 2%, and partial, 6.9%. The reoperation rate was 4% with a postoperative complication rate of 3.4%. Among these, there were: bleeding, 2.4%, which required in one case (0.5%) a surgical treatment, and pararectal hematoma, 1%, which was treated conservatively. Long-term complication rate was 6.9%, subdivided as follows: urgency, 2%; persistent bleeding, 2.5%; soiling, 1.4%; tenesmus, 0.5%; and painful suture, 0.5%.

### Group D

The recurrence rate was 4.3% and it was divided as complete, 0.7%, and partial, 3.6%. The reoperation rate was 1.4%. Early postoperative complication rate was 6.4%, including bleeding, 5.7%, requiring in one case a surgical revision, and urgency, 0.7%. The long-term complication rate was 2.8%, with urgency, 1.4%; stool incontinence, 0.7%; and soiling, 0.7%.

The success rate of all the procedures performed on patients selected through the new proposed A/CTC classification was higher in terms of trend of improvement (without any statistical significance *p* > 0.05), compared with the literature-based average success rate.

## Discussion

Nowadays, the introduction and diffusion of new techniques for HD surgical management have made it very clear how much Goligher historical classification is inadequate in the modern times, lacking in any correlation between anatomical and clinical features to a surgical procedure. Moreover, it may not reflect the true severity of the disease and its effects upon patients' quality of life. 10 The present study evaluates if the application of a new A/CTC classification proposed in the clinical practice correlates to a surgical outcomes' improvement.

Patients need to undergo the best possible treatment, which is the procedure that offers the best balance between minimally invasive surgery with less complications and greater therapeutic efficacy. Therefore, the correct indication may be achieved only through a proper classification and a correct therapeutic strategy that needs to consider all the following items: anatomic presentations, both pre- and intraoperative; type and frequency of symptoms with their impact on quality of life; contraindications due to concomitant comorbidities; and the associated diseases. In fact, in literature the HP is classified only according to its reducibility; 2 however, anatomy with prolapse dimension and characteristics represents an important parameter for therapeutic decision. In fact, THD, HAL-RAR, or TST may be enough to treat a small prolapse with well-detectable hemorrhoidal pedicle. Bigger and circumferential prolapses need, instead, a CSH. In case of irreducible HP with large and external piles, excisional hemorrhoidectomy is mandatory. Stapled procedures, in fact, are associated with a higher recurrence/persistence rate of skin tags and large hypertrophic external hemorrhoids 12 if compared with hemorrhoidectomy, 13 while THD and HAL-RAR reach the higher recurrence rate for fourth-degree hemorrhoids (11.1–59.3%). 14

The type and frequency of symptoms need to be considered to determine how effectively the HD affects patients' quality of life. Symptoms should be frequent or constant to support surgical indication in Groups B and C, but in Group D, there should be enough presence of recurrent acute hemorrhoidal crisis and/or complicated external component with edema (more than three times per year) to bear surgical indication. 15 16

Accurate and detailed symptoms assessment remains a milestone because some treatments proved to be more effective on specific symptoms, such as THD or HAL-RAR for bleeding 14 16 17 18 and CSH for HP. 19 20 21 Anyway, CSH resulted also to be effective in treating bleeding as major symptom. 20

The last parameter to be considered is the presence of associated diseases. Impaired anal continence can be considered a contraindication for CSH. In fact, after stapled procedure, urgency or anal incontinence may occur due to size reduction of the rectal ampulla and subsequent sensitivity alteration. 21 For these reasons, in case of preoperative impaired continence, THD, HAL-RAR, or hemorrhoidectomy are suggested. Excisional surgery is also indicated in case of IBD, to reduce risk of postoperative complications that is known to be higher in these patients. 22

Hemorrhoidectomy is also suggested in case of coagulation disorders or anticoagulant and/or antiplatelet therapy. In fact, a close technique such as CSH and THD or HAL-RAR could increase the risk of submucosal, intramural, or pararectal hematomas, 23 24 25 which may be life-threatening and more complex to manage than an external bleeding. Conversely, in case of concomitant ODS, a CSH with high-volume device is suggested because it can effectively treat both conditions due to rectal intussusception. 26

Regarding, instead, the comparison with the literature data, Group B showed a success rate consistent with the published data 20 but with lower reoperation rate. 27 In the present series, no perioperative and long-term complications were reported differently from published data with approximately 9% 27 and 20% 18 respectively of early and long-term complications rate.

In Group C, the success rate results to be higher if compared with the already published data ranging from 83.3 to 88.2% 27 with a considerably lower early and long-term postoperative complications rate. 19 28 29 30 31

The success rate of Group D is consistent with those reported about excisional surgery, around 95%, 32 with a lower postoperative complications and reoperation rates. 7 8 9 However, despite the published review, some long-term studies showed poor global success rates for all HD treatments, such as 65% for CSH, 9 73% for excisional surgery, and 70% for THD. 9 27 These contrasting results are consistent with the rate (19.1%) of previous hemorrhoidal surgery in the present series.

## Conclusion

The postoperative outcomes of all surgical procedures for HD were satisfactory if compared with the results reported in literature. These good results are probably due to a proper patient selection and also to an adequate surgical indication. In fact, A/CTC helped to ensure the surgeon to choose the best tailored treatment for each specific group of patients with the best balance between minimally invasive surgery and greater therapeutic efficacy. Hence, the proposed classification seems to improve the postoperative surgical outcomes.
